# Supplementary material for: Effect and process evaluation of a multicomponent public health intervention to increase the use of primary care-based mental health services amongst children with a migrant background in Stockholm, Sweden: a protocol for a mixed-methods study
Source: BMJ Open. 2021 Jul 14;11(7):e052820. doi: 10.1136/bmjopen-2021-052820 (PMC8280914; doi:10.1136/bmjopen-2021-052820)
Supplement: Author's manuscript [file bmjopen-2021-052820.draft_revisions.pdf]

BMJ Open is committed to open peer review. As part of this commitment we make the peer review history of every article we publish publicly available.

When an article is published we post the peer reviewers' comments and the authors' responses online. We also post the versions of the paper that were used during peer review. These are the versions that the peer review comments apply to.

The versions of the paper that follow are the versions that were submitted during the peer review process. They are not the versions of record or the final published versions. They should not be cited or distributed as the published version of this manuscript.

BMJ Open is an open access journal and the full, final, typeset and author-corrected version of record of the manuscript is available on our site with no access controls, subscription charges or pay-per-view fees (http://bmjopen.bmj.com).

If you have any questions on BMJ Open's open peer review process please email info.bmjopen@bmj.com

BMJ Open

Evaluation of short- and long-term effects and processes of a complex public health intervention to increase use of primary care-based mental health services amongst children with a migrant background: a study protocol.

Journal:	*BMJ Open*
Manuscript ID	bmjopen-2021-052820
Article Type:	Protocol
Date Submitted by the Author:	27-Apr-2021

Complete List of Authors:	Place, Vanessa; Karolinska Institutet, Department of Global Public Health<br>Assel, Karima; Karolinska Institutet, Department of Global Public Health<br>Hagström, Ana; Centrum för epidemiologi och samhällsmedicin, Unit for implementation and evaluation<br>Gubi, Ester; Karolinska Institutet, Department of Global Public Health<br>Augustsson, Hanna; Centre for Epidemiology and Community Medicine<br>Dalman, Christina; Karolinska Institutet, Department of Global Public Health<br>Hollander, Anna-Clara; Karolinska Institutet, Department of Global Public Health

Keywords:	MENTAL HEALTH, Protocols & guidelines < HEALTH SERVICES ADMINISTRATION & MANAGEMENT, Child & adolescent psychiatry < PSYCHIATRY

SCHOLARONE™
Manuscripts

I, the Submitting Author has the right to grant and does grant on behalf of all authors of the Work (as defined in the below author licence), an exclusive licence and/or a non-exclusive licence for contributions from authors who are: i) UK Crown employees; ii) where BMJ has agreed a CC-BY licence shall apply, and/or iii) in accordance with the terms applicable for US Federal Government officers or employees acting as part of their official duties; on a worldwide, perpetual, irrevocable, royalty-free basis to BMJ Publishing Group Ltd ("BMJ") its licensees and where the relevant Journal is co-owned by BMJ to the co-owners of the Journal, to publish the Work in this journal and any other BMJ products and to exploit all rights, as set out in our licence.

The Submitting Author accepts and understands that any supply made under these terms is made by BMJ to the Submitting Author unless you are acting as an employee on behalf of your employer or a postgraduate student of an affiliated institution which is paying any applicable article publishing charge ("APC") for Open Access articles. Where the Submitting Author wishes to make the Work available on an Open Access basis (and intends to pay the relevant APC), the terms of reuse of such Open Access shall be governed by a Creative Commons licence – details of these licences and which Creative Commons licence will apply to this Work are set out in our licence referred to above.

Other than as permitted in any relevant BMJ Author's Self Archiving Policies, I confirm this Work has not been accepted for publication elsewhere, is not being considered for publication elsewhere and does not duplicate material already published. I confirm all authors consent to publication of this Work and authorise the granting of this licence.

For peer review only

Evaluation of short- and long-term effects and processes of a complex public health intervention to increase use of primary care-based mental health services amongst children with a migrant background: a study protocol.

The names of the authors:

Vanessa Place[1], Karima Viksten Assel[1,2], Ana Hagström[3,4], Ester Gubi[1], Hanna Augustsson[3,4], Christina Dalman[1,4], Anna-Clara Hollander[1]

The affiliations of the authors, i.e. institution, department, city, state, country

1. Karolinska Institutet, Department of Global Public Health, Karolinska Stockholm (KI), Sweden

2. Transkulturellt Centrum, Stockholm, Sweden

3. Medical Management Centre, Department of Learning, Informatics, Management and Ethics, KI

4. Centre for Epidemiology and Community Medicine, Stockholm, Sweden

Corresponding author: vanessa.place@stud.ki.se

Word count: 4096

For peer review only

Abstract

Introduction: The overall aim of the project is to understand how to increase access to, and use of, primary care-based mental health services for children and youth from a migrant background with mild to moderate mental health problems.

Methods and analysis: The study will be undertaken in Haninge municipality in Stockholm, Sweden. The study has three intervention components: 1) A health communication intervention targeting parents of children/youth with a migrant background. 2) Training of professionals and volunteers who potentially have contact with parents and children with a migrant background, in order to increase the number of referrals to primary care-based mental health services. 3) Increasing access to care at a primary care-based mental health service for children, using various strategies to lower barriers to care. The complex multi-component intervention will be studied with an effect and a process evaluation methodology. Methodological limitations: the project is resource-intensive, thus a cost-benefit analysis would have been beneficial but was not feasible to include. In addition, there is a lack of available instruments in order to test these kinds of complex interventions, hence the research group use survey tools developed within the group in combination with previously tested questionnaires.

Ethics and Dissemination: All planned studied are approved by the Swedish Ethical Review Authority. The results of the project will be published continually in peer-reviewed scientific journals and disseminated to relevant stakeholders nationally and within Haninge municipality.

Article Summary

Strengths and limitations of this study

- A three-component complex public health intervention to increase use of primary care-based mental health services among children with a migrant background directed to different groups, including professionals and parents.
- A short- and long-term effect and process evaluation
- No cost-benefit analysis
- Limited to one municipality in Sweden
- The study is carried out during the Covid-19 pandemic, which could influence the number of staff at the primary care-based mental health services and has transferred the interventions online, which could influence the results.

For peer review only

Introduction

In the Scandinavian universal health care context several studies indicate that, despite higher need of psychiatric care[1-4], there is a lower psychiatric service utilisation among all migrants[5] including migrant children and youth (both refugees and other) compared their peers[6-8]. The level of psychiatric care utilisation among migrant children is particularly low in primary care-based mental health services[6] and specialist outpatient care as compared to emergency psychiatric care[6, 9]. Studies have indicated that utilisation of psychiatric care is lower amongst migrant youth during their first years in their new country, but that levels do increase over time[8]. The reasons behind the underutilisation of psychiatric services among children with a migrant background are not yet well understood, but a wide range of barriers to mental healthcare are likely to contribute[10]. International studies suggest that the barriers to mental healthcare amongst migrant children and youth are generally similar to those faced by their peers[10] but that migrant children in addition face additional barriers linked to their minority status[10]. Overcoming general as well as migration-specific barriers will be critical to meeting the mental health needs of migrant children and youth.

There are very few studies on interventions to overcome barriers to mental health services for migrant children. Research has, however, indicated that schools and social agencies play an important role in referring children from migrant backgrounds to mental health services[11]. Additionally, a scoping review mapping the interventions to increase care-seeking amongst adult migrant groups for stigmatised conditions, including mental health, concluded that health communication strategies and complex, multi-component interventions were promising strategies[12] whereas one-component interventions (translating information materials, for instance) had little effect[12].

In the absence of scientifically evaluated methods to overcome barriers to mental health services faced by migrant children, local models have developed. Since 2010, the Stockholm Region's centre for migration and health, Transkulturellt centrum (www.transkulturelltcentrum.se/eng) has offered regular training to healthcare staff on migration, health and transcultural issues, as well as group-based health communication (henceforth called health communication) to migrants in their mother tongue. Health communication is run by individuals with a medical or public health background.

Drawing together the available evidence on barriers to care, we have designed a multi-component complex public health intervention study that targets, in parallel: key professionals and volunteers, parents with a migrant background, and the structural barriers faced when contacting mental health services. Given the low use of primary care-based mental health services in Stockholm and the potential benefits of early interventions, the 'Bridging barriers to care for children with a migrant background' (given the acronym SAM-TINA in Swedish) research group (referred to herein as the research group) seeks to improve access to, and use of, primary care-based mental health services for children with a migrant background.

Study aims and objectives

For the purposes of the project, migrant background is defined as children under 18 years of age who have themselves, or both of their parents, been in Sweden for less than ten years.

The overall aim of the project is to investigate how to increase access to, and use of, care from primary care-based mental health services for children and youth with a migrant background with mild to moderate mental health problems.

Methods and analysis

Site

For peer review only - http://bmjopen.bmj.com/site/about/guidelines.xhtml

The study will be undertaken in Haninge municipality in Stockholm, Sweden. In 2020, Haninge had a population of 93 282, 28% of whom were born outside Sweden (compared to 19,7% in Sweden as a whole). 11% of 0-17 year olds in Haninge in 2020 were born outside Sweden.

The primary care-based mental health service in focus is Psykosociala mottagningen at the primary care centre Aleris Rudan (https://www.aleris.se/har-finns-vi/stockholm/aleris-rudans-vardcentral/) in Haninge, herein referred to as "Rudan" and "the primary care centre", respectively. Rudan is the second largest primary care-based mental health service by number of visits in Stockholm County. Despite many new patients, and the high proportion of the population in the catchment area who are foreign-born, Rudan has received very few patients with a migrant background in recent years.

<u>The three-component intervention</u>

For an overview of the three-component intervention and the effect and process evaluation, see Figure 1.

SAM-TINA has three components:

Component 1: A health communication intervention targeting parents of children/youth with a migrant background.

Component 1 in detail:

The health communication intervention will be delivered in one session by health communicators, either at a venue (see 'Planning and preparations for Component 1') or digitally (in line with restrictions due to the Covid-19 pandemic). The health communication sessions are offered to parents of children and youth with a migrant background and are delivered over a period of 6-12 months. Each session is approximately 2.5 hours long, with a minimum of 2-3 participants and a maximum of 8 participants. Sessions are delivered in participant's mother tongue (Arabic, Somali, Tigrinya, Amarinya, or Farsi), in simple Swedish, or with an interpreter. Sessions are offered primarily in languages deemed most common in the community by staff members at venues hosting the sessions. Sessions in other languages are offered upon active interest from participants.

Planning and preparing for Component 1:

In 2019-2020, the research group and Transkulturellt Centrum worked to create partnerships with units of the municipality and non-profit organisations within Haninge. Suitable hosts and venues for were identified with the help of the municipality's coordinators for refugee integration and parental support, "Swedish for Immigrants" classes in Haninge, parental support groups run by the local municipality targeting newly-arrived parents, toddler-parent groups, and the Swedish Church. Recruitment, group scheduling, and later Covid-19 adjustments were planned in joint meetings between these stakeholders, Transkulturellt Centrum, and SAM-TINA researchers. Additional venues may be added during the study.

Workshops with health communicators at Transkulturellt Centrum aimed to increase their knowledge of child mental health and the organization of mental health services. These workshops were led by an experienced child psychologist and child psychiatrist. In addition, a specific tool kit was developed, in Swedish, consisting of PowerPoint slides with discussion points and case discussions, covering six chapters:

I. Normal development compared with signs of mental distress among children and adolescents
II. Parenting skills
III. Potential barriers to care
IV. Available mental health services and care levels
V. Services at Rudan and the role of a psychologist
VI. How to contact Rudan

For peer review only

Evaluation measures of Component 1

- Follow up questionnaires will be distributed before, directly after, and three months after the intervention by post, e-mail, or by hand to parents who have signed up for the sessions and consented to be contacted. In the absence of a validated instrument, this questionnaire was created for SAM-TINA. All questionnaires have been translated to the language the session was delivered in.
- A questionnaire for patients visiting Rudan for the first time will be given out to all new visitors, by email or by hand. The questionnaire explores why visitors chose to seek help at Rudan and how they accessed the service. For patients younger than 14 years old, a caregiver will be asked to fill in the questionnaire, if present.
- Analysis of register data from multiple registers, including the Psychiatry Sweden database held by the research group EPiCSS (https://ki.se/en/gph/epidemiology-of-psychiatric-conditions-substance-use-and-social-environment-epicss), as done in [5-7].
- Focus group interviews, see 'The process evaluation of the three-component intervention' below.

Component 2: Training for professionals and volunteers (herein referred to as TINA-facilitators) who potentially have contact with parents and children with a migrant background, in order to increase referrals to Rudan.

Component 2 in detail:

Training of TINA-facilitators is implemented and moderated by Transkulturellt Centrum's staff (except for the volunteer training, which is organised by the research group). The course length and format varies, depending on participating organisation's ability to allow staff to attend. All trainings are delivered digitally to minimise the spread of Covid-19. Most are one-day sessions, but can be two half-day or three short sessions. Online training for pre-school teachers, with pre-recorded material, is accessible for 4-6 weeks.

Planning and preparing for Component 2:

The research group mapped the demographics of the Haninge municipality (see 'Site'), including a mapping of the number of children with a migrant background by residential area, in order to identify priority neighbourhoods for the intervention. Four neighbourhoods were identified. In 2019-2020, the research group and Transkulturellt Centrum worked to recruit healthcare, municipality and community organisations in Haninge. We primarily invited units serving children in the four areas to participate in the training intervention. To tailor the content, format, and duration of the trainings to the diverse needs of the participating units, nine group interviews were conducted. Based on the results of these interviews and Transkulturellt Centrum´s previous experience with providing staff training, seven separate training programs were designed. All are a minimum of four hours with a common core of lectures on:

i. How to identify mental distress among children and adolescents
ii. Potential mental health consequences of migration
iii. Barriers to care for migrants in Sweden
iv. Mental health services at Rudan

Moreover, lectures were added to cover specific needs of the individual units, such as how to differentiate learning disabilities from post-traumatic stress for the school healthcare staff. After the training, all participants will be offered information digitally or on paper about Rudan (in Polish, Russian, Arabic, Turkish, Spanish, Somali, Tigrinya, English, French, Persian/Dari, and Swedish) to distribute to eligible families.

For peer review only

Evaluations measures of Component 2

- Questionnaires, including short-term effect and process outcomes, will be sent by email to TINA facilitators who signed up for the training and have consented to be contacted before, directly after, and three months after the training. These questionnaires are a combination of a modified version of a questionnaire previously used in the region for similar purposes,[13] and items on readiness for change from the implementation outcome measure (IPM)[14]. They also include questions from a previously used survey [15] to measure learning and reactions to evaluate the training experience.
- A similar, shorter version of the Component 2 questionnaire has been developed for the volunteer training.
- The questionnaire to first-time patients at Rudan, see 'Evaluations measures of Component 1'.
- Analysis of register data, see 'Evaluations measures of Component 1'.
- Semi-structured interviews, see 'The process evaluation of the three-component intervention'.

Component 3: Increasing access to care at Rudan through various strategies to lowering barriers to seeking care.

Planning and preparing for Component 3:

Interviews with directors and staff at primary care-based mental health services for children were conducted to explore barriers to care and support given to migrant parents (published in Swedish)[16]. The research group then worked with Rudan's manager and the head of the primary health-care centre to anchor the project, and a project coordinator was appointed at Rudan. Through discussions with the project coordinator, staff at the primary health centre, Rudan's psychologists, and Transkulturellt Centrum's health communicators, the research group suggested the following interventions to lower barriers to care for migrant families:

- Written information (leaflets, posters, and business cards) about Rudan available in the primary healthcare centre waiting area and its website (https://www.aleris.se/har-finns-vi/stockholm/aleris-rudans-vardcentral/psykosocial-mottagning-barn-och-ungdom/) in Polish, Russian, Arabic, Turkish, Spanish, Somali, Tigrinya, English, French, Persian/Dari, and Swedish.
- A direct telephone number for appointment booking, without a phone menu, available from March 2021. The phoneline is manned from 14:00-15:00 during the clinic's regular opening hours. Messages left outside of these hours will be listened to once a day and individuals contacted.
- Appointment booking drop-in service at Rudan's reception available from March 2021. Drop-in is available between 14:00-15:00 during regular opening hours.
- A tablet available in Rudan's reception, with translation provided by the app Care-to-Translate (https://caretotranslate.com/sv/), for appointment booking from April 2021.
- A leaflet sent out to all new patients at Rudan, together with appointment details and in the child's or parent/caregiver's preferred language, containing information on what to expect from their first visit. Available from June 2021.

Evaluations measures of Component 3

- Questionnaire to first-time patients at Rudan, see 'Evaluations measures of Component 1'.
- Analysis of register data, see 'Evaluations measures of Component 1'.
- A daily log, kept by the project coordinator, to document any implementation issues.
- Interviews with Rudan's staff to explore challenges in the implementation process and to understand barriers and facilitators for implementation.

Time frame:

The three components are implemented in parallel in order to create a synergistic effect:

Component 1: Trainings are organised over a period of 5.5 months (start date: Nov 11th 2020, planned completion: May 30th 2021).

Component 2: Health communication sessions will be offered over a 6–12-month period (planned start in Nov 2020 was delayed until Feb 2021).

Component 3: Interventions will be implemented over a 12-month period from March 2021.

Recruitment and eligibility

Component 1: Health communication interventions targeting parents of children/youth with a migrant background.

Recruitment of participants

Recruitment of parents is currently ongoing. Information in multiple languages is being disseminated to parents by health communicators and hosts at the venues (see 'The three-component intervention'). In addition, short video clips in Arabic, Tigrinya, Somali, Farsi and easy Swedish have been developed for the project and will be disseminated online. They can be viewed at the project homepage (https://ki.se/gph/informationstraffar-for-foraldrar-i-haninge).

Eligibility

All parents to minors are eligible to take part, independent of country of birth, length of stay in Sweden, or language. However, a minimum of 2-3 participants are required for a session, which could lead to exclusion of parents with a less commonly spoken preferred language. All participants are welcome in the sessions, independent of literacy level, but illiterate participants are excluded from the follow-up evaluations due to the nature of the questionnaire.

Component 2: Training of TINA-facilitators who potentially have contact with parents and children with a migrant background, to increase the number of referrals to Rudan.

Recruitment of participants

Recruitment for the TINA facilitator training for professionals was achieved mainly with the help of key staff members, unit directors, and posts on the municipality's intranet. Some of the training programs were advertised in Transkulturellt Centrum's overall training program which reaches healthcare staff within the Stockholm Region. The recruitment of participants for the TINA -facilitator training for volunteers engaged in civil society with Haninge is still ongoing, assisted by key stakeholders and contact with community organisations.

Eligibility

Individuals who are expected to have contact with children, adolescents, and families with a migrant background are eligible to participate. This includes staff from the primary healthcare centres, paediatric clinics, adolescent health and addiction centres, social services, pre-schools, schools, and non-profit organisations (such as sports teams). Units who serve the populations in the four prioritised neighbourhoods (see 'Site') were prioritised for recruitment.

Component 3: Increasing access to care at Rudan through various strategies to lowering barriers to seeking care at this service.

Recruitment of participants and eligibility

For peer review only

All children under 18 years of age who have themselves, or both of their parents, been in Sweden for less than ten years and who have visited the primary healthcare centre are eligible to participate.

<u>The effect evaluation of the three-component intervention</u>

An effect evaluation aims to establish the causal association between an intervention in the target population and its intended outcomes.

Primary aim

The primary aim of the effect evaluation is to assess if the three components together increase the number of children seeking care at Rudan for mild to moderate mental health problems for the first time.

Research questions

1. Will the observed rate of children seeking care at Rudan for the first time exceed the expected during the 12 months following start of the intervention (exposed period), based on the rate of access in the 24 months preceding the intervention (unexposed period)?
2. Will there be a change in the demographic composition of children seeking care at Rudan following start of the intervention (e.g. own or parents´ country/region of birth, time in Sweden, migrant status, and age)?
3. Will the observed rate and demographic composition of children seeking care at Rudan for the first time during the 12 months following start of the intervention, based on the rate of the 24 months preceding the intervention, differ as compared to other primary-based mental health services in the Stockholm Region?

Outcome measure

Number of first visit consultations at Rudan (onsite and digital) before and 3-, 6- and 12-months following start of interventions.

Secondary aim

The secondary aim of the effect evaluation is to investigate if the three individual components work as they were intended to.

Research questions

a. Will there be a change in the self-perceived knowledge and attitudes among staff and/or volunteers exposed to the training component? (e.g. increase in knowledge of signs of mental distress and change in attitudes towards working with families with a migrant background)?
b. Will the observed rate of referrals to Rudan from units exposed to the training exceed the expected during the 12 months following start of the training component, based on the rate of access in the 24 months preceding the intervention), and differ compared to other unexposed units?
c. Will there be a change in the self-perceived knowledge and attitudes among parents exposed to the health communication component (e.g increased knowledge of mental health)?
d. What is the self-reported pathways to seek the service among first-time patients at Rudan following the start of the intervention?
e. Will there be a change in perceived barriers to connect with the service, among first-time patients at Rudan following start of the Component 3?

Outcome measures

For peer review only

- Staff and volunteer self-reported knowledge and attitudes at baseline, directly after, and 3 months following the training component (measured using the questionnaire for staff and the questionnaire for volunteers).
- Parents self-reported knowledge and attitudes at baseline, directly after, and 3 months following the health communication component (measured using the health communication questionnaire).
- First-time patients self-reported pathway to Rudan and self-perceived barriers to seek care at the service (using the first visit questionnaire).
- Number of referrals to Rudan from units exposed to the training component at baseline and 3-, 6-, and 12-months following start of the training (measured using data extracted from registers, see 'Primary aim: outcome measures' and from the first-visit questionnaire).

Methods and statistical analyses

For the primary aim we will analyse changes over time in children seeking care at Rudan and other primary-care based services in Stockholm before and following start of intervention using the linked register data (as done in [5-7]) in a time-series design, where the outcome will be explored in one-month units, using Poisson regressions. This method will also be used to analyze one of the secondary aims: referrals to Rudan. For the other secondary aims we will analyze the questionnaires using multiple logistic regressions models in both cross sectional and longitudinal designs.

<u>The process evaluation of the three-component intervention</u>

The process evaluation aims to facilitate an understanding of the effects of the intervention and provide information about under which circumstances a complex public health intervention to increase help-seeking among migrants can be implemented. The process evaluation will focus on assessing implementation fidelity (i.e. adherence to the intervention, and moderating factors that may influence the degree of fidelity) using a modified version of the Conceptual Framework for Implementation Fidelity (CFIF)[17].

Data collection and participants

A mixed methods approach will be applied to collect data, including questionnaires, semi-structured interviews, focus group discussions and diaries. Interview guides will be developed based on the guiding framework, CFIF[17]. The interviews and focus group discussions will be conducted and reported following the COREQ checklist[18]. The data collection for each intervention component is outlined below and in Table 1.

Component 1: A health communication intervention targeting parents of children/youth with a migrant background.

Questionnaires at baseline, directly after, and three months after the health communication session will be used to assess satisfaction, usefulness, and benefits of the sessions, as well as to understand the demographics of parents who were recruited and attended the sessions.

We will perform focus group discussions with health communication participants in order to get a deeper understanding of their perceptions of the sessions. These groups will be conducted in the preferred (Arabic, Dari, Somali and Tigrinya) language of the participants, and the interview guide will be translated from Swedish to this language before back-translation to Swedish to check for accuracy. Recruitment of participants for the focus group discussions will be conducted by the key actors at the venues recruiting participants to the health communication. A purposeful sampling approach will be used to ensure variation in the sample (including participants from different countries). The focus group discussions will be conducted in parallel to the health communication sessions and will be ongoing throughout this component of the intervention.

For peer review only

Component 2: Training of TINA-facilitators who potentially have contact with parents and children with a migrant background, to increase the number of referrals to Rudan.

The training of professionals will be evaluated by combining data from distributed questionnaires at baseline, directly after, and three months after the training, as well as interviews. Attendance lists will be used to collect information about the number of recruited participants and what proportion of target group participated in this component (coverage).

Semi-structured interviews will be conducted to assess participants' satisfaction with the training, whether the training led to behaviour changes, and potential moderating factors to implementation of what was learned from the training at the workplace. To understand contextual and organisational differences, a purposive sampling approach will be applied to recruit informants from different work units and professional groups. During the training, participants are asked to reflect on how to transfer knowledge gained during the training to their practice, and to concretise this into a plan for how to identify and refer children with mental health problems to the correct care level. The evaluation team will follow up on if the plan has been created and implemented by contacting an assigned "contact person" at the participating units. The contact persons will be interviewed and, if necessary, more participants will be invited for interviews to assess the above-mentioned implementation outcomes.

Component 3: Increasing access to care at Rudan by various strategies to lower barriers to seeking care.

A notebook will be used by staff at Rudan to document foreseen and unforeseen events related to the interventions put in place related to the third intervention component. The notebook is a register of events and processes that take place over time. It will also be used for documenting problems that arise, and to note impressions and experiences of what happened.

Data Analysis

The use of mixed methods will allow quantitative and qualitative combination of data collection sources which can validate the findings and give a deeper understanding of the implementation process.

- Qualitative analysis

All interviews and focus group discussions will be audio recorded and transcribed verbatim. The transcripts will be analysed using deductive content analysis[19] based on the guiding framework CFIF. Additional qualitative data sources are information documented in the diary. Reported expected and unintended events during the period of implementation will be compiled and added to the analysis.

- Statistical analysis

For survey data, statistical analyses will be used to measure changes over time within the groups of training participants and health communication participants. Data on moderating factors and fidelity will be used to predict intervention outcomes, (for example increased knowledge as assessed in the effect evaluation). Survey answers will be dichotomised (yes/no) and logistic regression or mixed logistic regression models will be used to explore possible variation over time. As sensitivity analyses, ordinal logistic regression will be performed to test the association for ordinal outcomes as a 5-score scale. Possible heterogeneity among health centres will be taken into account using multilevel models.

<u>Methodological limitations</u>

SAM-TINA is resource-intensive, so a cost-benefit analysis would have been beneficial but was not feasible. Furthermore, a lack of available instruments to evaluate complex interventions meant that

For peer review only

the research group developed the survey tools themselves. Finally, the fact that the intervention included just one municipality may limit the generalisability of findings to other contexts.

Patient and Public Involvement

Haninge municipality and non-profit organisations within Haninge were involved in the research from the planning stage (see 'Planning and preparing for Component 1-3'). Their involvement helped us to plan and design the components but not in their evaluation; this was carried out solely by the research group (with the exception of testing the pilot versions of the questionnaires). The findings will be disseminated to relevant stakeholders within Haninge municipality and nationally.

Ethics and Dissemination

All planned studied are approved by the Swedish Ethical Review Authority dnr 2017-135-31/5, 2019-06275, 2020-03640, 2020-06341, 2020-03642 and 2020-04180.

Informed consent will be obtained from all participants. The results of the project will be published continually in peer reviewed scientific journals (four articles planned) and presented at conferences over a minimum period of eight years. Beyond academic output, the findings of the SAM-TINA project will be disseminated to relevant stakeholders within Haninge municipality and nationally.

Figure Legends

Table 1. The general process evaluation plan including general process outcome questions, areas to measure and data sources.

Figure 1. A schematic overview of the three component intervention and the effect and process evaluation.

References

1. Kouider EB, Lorenz AL, Dupont M, Petermann F. Anxiety disorders in migrant and nonmigrant children: Risk factors and treatment success. *Kindh Entwickl*. 2015;24:252-262
2. Barghadouch A, Kristiansen M, Jervelund SS, Hjern A, Montgomery E, Norredam M. Refugee children have fewer contacts to psychiatric healthcare services: An analysis of a subset of refugee children compared to danish-born peers. *Social Psychiatry and Psychiatric Epidemiology*. 2016;51:1125-1136
3. Lindstrom M, Moden B, Rosvall M. Country of birth, parental background and self-rated health among adolescents: A population-based study. *Scandinavian Journal of Public Health*. 2014;42:743-750
4. Bronstein I, Montgomery P, Dobrowolski S. Ptsd in asylum-seeking male adolescents from afghanistan. *J Trauma Stress*. 2012;25:551-557
5. Hollander AC, Mackay E, Sjoqvist H, Kirkbride JB, Baarnhielm S, Dalman C. Psychiatric care use among migrants to sweden compared with swedish-born residents: A longitudinal cohort study of 5 150 753 people. *BMJ Glob Health*. 2020;5
6. Sirén A, Wicks S, Lindberg L, Dalman C. Uppföljning av första linjen psykisk ohälsa barn och unga 2018
7. Axelsson L, Baarnhielm S, Dalman C, Hollander AC. Differences in psychiatric care utilisation among unaccompanied refugee minors, accompanied migrant minors, and swedish-born minors. *Soc Psychiatry Psychiatr Epidemiol*. 2020;55:1449-1456
8. Manhica H, Almquist Y, Rostila M, Hjern A. The use of psychiatric services by young adults who came to sweden as teenage refugees: A national cohort study. *Epidemiol Psych Sci*. 2017;26:526-534
9. Bjorkenstam E, Helgesson M, Norredam M, Sijbrandij M, de Montgomery CJ, Mittendorfer-Rutz E. Differences in psychiatric care utilization between refugees, non-refugee migrants and swedish-born youth. *Psychol Med*. 2020:1-11

For peer review only

10. Place V, Nabb B, Assel K, Åhlen J, Gubi E, Hagström A, et al. Perceived barriers to care for migrant children and young people with mental and neurodevelopmental health problems in high-income countries: A meta-ethnography. Submitted
11. Fazel M, Reed RV, Panter-Brick C, Stein A. Mental health of displaced and refugee children resettled in high-income countries: Risk and protective factors. *Lancet*. 2012;379:266-282
12. Place V, Nabb B, Viksten Assel K, Baarnhielm S, Dalman C, Hollander AC. Interventions to increase migrants' care-seeking behaviour for stigmatised conditions: A scoping review. *Soc Psychiatry Psychiatr Epidemiol*. 2021
13. Baarnhielm S, Edlund AS, Ioannou M, Dahlin M. Approaching the vulnerability of refugees: Evaluation of cross-cultural psychiatric training of staff in mental health care and refugee reception in sweden. *BMC Med Educ*. 2014;14:207
14. Randall R, Nielsen K, Tvedt SD. The development of five scales to measure employees' appraisals of organizational-level stress management interventions. *Work Stress*. 2009;23:1-23
15. Mosson R, Augustsson H, Back A, Ahstrom M, Schwarz UV, Richter A, et al. Building implementation capacity (bic): A longitudinal mixed methods evaluation of a team intervention. *Bmc Health Services Research*. 2019;19
16. Åhlén J, Dalman C. Barn och ungdomar med migrationsbakgrund i första linjen för psykisk ohälsa: Tillgänglighet till vård, tillgång till översatta arbetsinstrument, och verksamhetens struktur. En rapport inom tina-projektet. . 2020
17. Hasson H. Systematic evaluation of implementation fidelity of complex interventions in health and social care. *Implementation Science*. 2010;5
18. Tong A, Sainsbury P, Craig J. Consolidated criteria for reporting qualitative research (coreq): A 32-item checklist for interviews and focus groups. *Int J Qual Health C*. 2007;19:349-357
19. Hsieh HF, Shannon SE. Three approaches to qualitative content analysis. *Qualitative Health Research*. 2005;15:1277-1288

For peer review only

Table 1. The general process evaluation plan including general process outcome questions, areas to measure and data sources.

Process evaluation objective	Overarching research questions	Data source and data collection
**Adherence**		
**Content**	Were the three intervention components implemented as planned?	Questionnaires, interviews, diary
**Coverage**	What proportion of the targeted groups participated in the intervention?	Attendance lists
**Moderating factors**		
**Recruitment**	What barriers for maintaining involvement of individuals/groups in the intervention was identified?<br>What recruitment procedures were used to engage professionals and parents in the intervention?	Meeting minutes, interviews, surveys
**Participant responsiveness**	Did participants find intervention content useful?<br>How satisfied were the participants with the intervention services?<br>How did the participants perceive the relevance of the intervention?	Questionnaires, interviews
**Strategies to facilitate implementation**	Which strategies were put in place by work units participating in the training of professionals to implement learnings from the training?	Interviews
**Context (inner and outer)**	What factors at policy, organisational and<br>work group levels affected the implementation?<br>Which barriers and facilitators to delivery as planned can be identified?	Interviews, Meeting minutes, Diary
**Quality of delivery**	How did professionals perceive the impact of the intervention e.g., which elements did they find to be most helpful and unhelpful in meeting their needs?	Interviews
**Intervention complexity**	How complex is this intervention?	A group of external researchers will evaluate the intervention complexity

Modified from Hasson H. Systematic evaluation of implementation fidelity of complex interventions in health and social care [17].

For peer review only

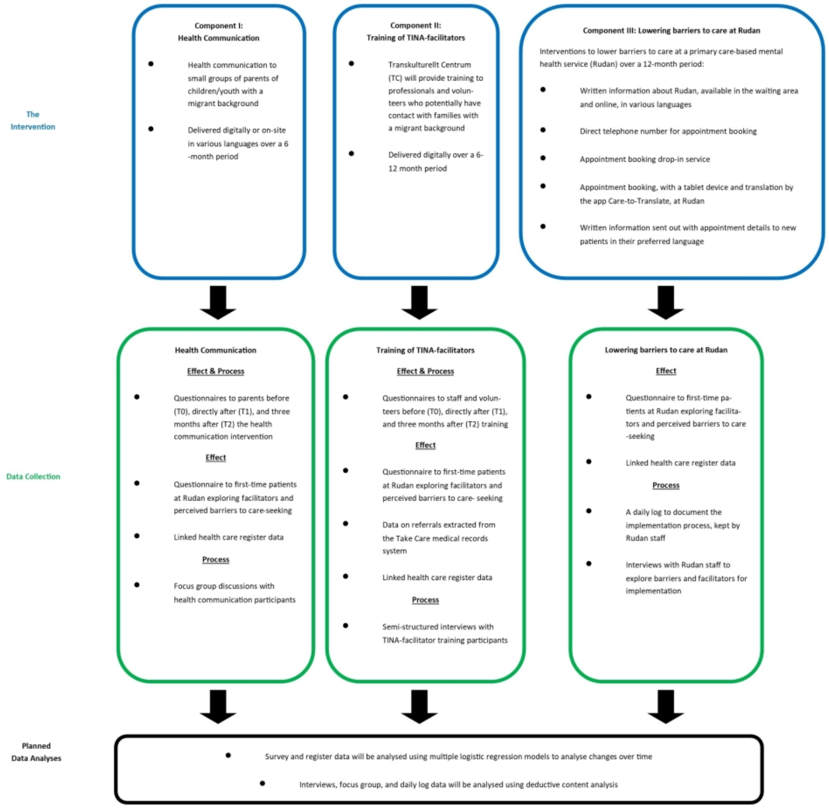

Figure 1. A schematic overview of the three component intervention and the effect and process evaluation.

209x296mm (300 x 300 DPI)

BMJ Open

Effect and process evaluation of a multi-component public health intervention to increase the use of primary care-based mental health services amongst children with a migrant background in Stockholm, Sweden: a protocol for a mixed-methods study.

Journal:	*BMJ Open*
Manuscript ID	bmjopen-2021-052820.R1
Article Type:	Protocol
Date Submitted by the Author:	01-Jul-2021
Complete List of Authors:	Place, Vanessa; Karolinska Institutet, Department of Global Public Health<br>Assel, Karima; Karolinska Institutet, Department of Global Public Health<br>Hagström, Ana; Karolinska Institute, Medical Management Centre, Department of Learning, Informatics, Management and Ethics<br>Gubi, Ester; Karolinska Institutet, Department of Global Public Health<br>Augustsson, Hanna; Karolinska Institute, Medical Management Centre, Department of Learning, Informatics, Management and Ethics<br>Dalman, Christina; Karolinska Institutet, Department of Global Public Health<br>Hollander, Anna-Clara; Karolinska Institutet, Department of Global Public Health
**Primary Subject Heading**:	Public health
Secondary Subject Heading:	Mental health, Public health
Keywords:	MENTAL HEALTH, Protocols & guidelines < HEALTH SERVICES ADMINISTRATION & MANAGEMENT, Child & adolescent psychiatry < PSYCHIATRY

SCHOLARONE™
Manuscripts

I, the Submitting Author has the right to grant and does grant on behalf of all authors of the Work (as defined in the below author licence), an exclusive licence and/or a non-exclusive licence for contributions from authors who are: i) UK Crown employees; ii) where BMJ has agreed a CC-BY licence shall apply, and/or iii) in accordance with the terms applicable for US Federal Government officers or employees acting as part of their official duties; on a worldwide, perpetual, irrevocable, royalty-free basis to BMJ Publishing Group Ltd ("BMJ") its licensees and where the relevant Journal is co-owned by BMJ to the co-owners of the Journal, to publish the Work in this journal and any other BMJ products and to exploit all rights, as set out in our licence.

The Submitting Author accepts and understands that any supply made under these terms is made by BMJ to the Submitting Author unless you are acting as an employee on behalf of your employer or a postgraduate student of an affiliated institution which is paying any applicable article publishing charge ("APC") for Open Access articles. Where the Submitting Author wishes to make the Work available on an Open Access basis (and intends to pay the relevant APC), the terms of reuse of such Open Access shall be governed by a Creative Commons licence – details of these licences and which Creative Commons licence will apply to this Work are set out in our licence referred to above.

Other than as permitted in any relevant BMJ Author's Self Archiving Policies, I confirm this Work has not been accepted for publication elsewhere, is not being considered for publication elsewhere and does not duplicate material already published. I confirm all authors consent to publication of this Work and authorise the granting of this licence.

For peer review only

Effect and process evaluation of a multi-component public health intervention to increase the use of primary care-based mental health services amongst children with a migrant background in Stockholm, Sweden: a protocol for a mixed-methods study.

The names of the authors:

Vanessa Place[1], Karima Viksten Assel[1,2], Ana Hagström[3,4], Ester Gubi[1], Hanna Augustsson[3,4], Christina Dalman[1,4], Anna-Clara Hollander[1]

The affiliations of the authors, i.e. institution, department, city, state, country

1. Karolinska Institutet, Department of Global Public Health, Karolinska Stockholm (KI), Sweden

2. Transkulturellt Centrum, Stockholm, Sweden

3. Medical Management Centre, Department of Learning, Informatics, Management and Ethics, KI

4. Centre for Epidemiology and Community Medicine, Stockholm, Sweden

Corresponding author: Vanessa Place, vanessa.place@stud.ki.se, research group: Epidemiology of Psychiatric Conditions, Substance use and Social environment (EPICSS), Department of Public Health Sciences, Karolinska Institutet, Solnavägen 1E, SE-171 77, Stockholm, phone number: +46707567136

Word count: 4234

For peer review only

Abstract

Introduction: The overall aim of the project is to understand how to increase access to, and use of, primary care-based mental health services for children and youth from a migrant background with mild to moderate mental health problems.

Methods and analysis: The study will be undertaken in Haninge municipality in Stockholm, Sweden. The study has three intervention components: 1) A health communication intervention targeting parents of children/youth with a migrant background. 2) Training of professionals and volunteers who potentially have contact with parents and children with a migrant background, in order to increase the number of referrals to primary care-based mental health services. 3) Increasing access to care at a primary care-based mental health service for children, using various strategies to lower barriers to care. The complex multi-component intervention will be studied with an effect and a process evaluation methodology.

Ethics and Dissemination: All planned studied are approved by the Swedish Ethical Review Authority. The results of the project will be published continually in peer-reviewed scientific journals and disseminated to relevant stakeholders nationally and within Haninge municipality.

Article Summary

Strengths and limitations of this study

- This multi-component public health intervention includes a short- and long-term effect and process evaluation, using a range of methods including pre- and post-surveys, analysis of register data from multiple registers, and individual and focus group interviews.
- The intervention, through both language-specific and more general components, targets both youth and parents from a migrant background, and professionals and volunteers who potentially have contact with this group.
- The study is being conducted during the Covid-19 pandemic, which has transferred the interventions online and could influence staffing levels at the primary care-based mental health services.
- The lack of available instruments to evaluate complex interventions led the research group to develop their own survey tools, in combination with previously tested questionnaires, which could reduce the reliability of findings.
- Due to its resource intensity, the project was limited to one municipality in Sweden, which may limit the generalisability of its findings to other contexts.

For peer review only

Introduction

In the Scandinavian universal healthcare context, studies indicate that despite higher need of psychiatric care [1-4], there lower psychiatric service utilisation amongst all migrants [5], including migrant children and youth, compared to their peers [6-8]. The level of psychiatric care utilisation among migrant children is particularly low for primary care-based mental health services [6] and specialist outpatient care when compared to emergency psychiatric care [6,9]. Studies have indicated that utilisation of psychiatric care is lower amongst migrant youth during their first years in their new country, but that levels do increase over time [8]. The reasons behind the underutilisation of psychiatric services among children with a migrant background are not yet well understood, but a wide range of barriers to mental healthcare are likely to contribute [10]. International studies suggest that the barriers to mental healthcare amongst migrant children and youth are generally similar to those faced by their peers [10], but that migrant children face additional barriers linked to their minority status [10]. Overcoming general, as well as migration-specific, barriers will be critical to meeting the mental health needs of migrant children and youth.

There are few studies on interventions to overcome barriers to mental health services for migrant children. Research has, however, indicated that schools and social agencies play an important role in referring children from migrant backgrounds to mental health services [11]. Additionally, a scoping review mapping the interventions to increase care-seeking amongst adult migrant groups for stigmatised conditions, including mental health, concluded that health communication strategies and complex, multi-component interventions were promising strategies [12] whereas one-component interventions (such as translating information materials) had little effect [12].

In the absence of scientifically evaluated methods to overcome barriers to mental health services faced by migrant children, local models have developed. Since 2010, the Stockholm Region's centre for migration and health, Transkulturellt centrum (www.transkulturelltcentrum.se/eng) has offered regular training to healthcare staff on migration, health and transcultural issues, as well as group-based health communication (henceforth called health communication) to migrants in their mother tongue. Health communication is run by individuals with a medical or public health background.

Drawing together the available evidence on barriers to care, we have designed a multi-component complex public health intervention study that targets, in parallel: key professionals and volunteers, parents with a migrant background, and the structural barriers faced when contacting mental health services. Given the low use of primary care-based mental health services in Stockholm and the potential benefits of early interventions, the 'Bridging barriers to care for children with a migrant background' (given the acronym SAM-TINA in Swedish) research group (referred to herein as the research group) seeks to improve access to, and use of, primary care-based mental health services for children with a migrant background.

Study aims and objectives

For the purposes of the project, migrant background is defined as children under 18 years of age who have themselves, or both of their parents, been in Sweden for less than ten years.

The overall aim of the project is to investigate how to increase access to, and use of, care from primary care-based mental health services for children and youth with a migrant background with mild to moderate mental health problems.

Methods and analysis

Site

The study will be undertaken in Haninge municipality in Stockholm, Sweden. In 2020, Haninge had a population of 93 282, 28% of whom were born outside Sweden (compared to 19.7% in Sweden as a whole). 11% of 0-17 year olds in Haninge in 2020 were born outside Sweden. The share of children who have themselves, or both of their parents, been in Sweden for less than ten years (our target population) will be calculated as part of the effect evaluation. However, as Swedish registers do not record race, culture, or ethnicity data (only country of origin) the proportions of different cultural backgrounds will not be calculated.

The primary care-based mental health service in focus is Psykosociala mottagningen at the primary care centre Aleris Rudan (https://www.aleris.se/har-finns-vi/stockholm/aleris-rudans-vardcentral/) in Haninge, herein referred to as "Rudan" and "the primary care centre", respectively. Rudan is the second largest primary care-based mental health service, by number of visits, in Stockholm County. Despite many new patients, and the high proportion of the population in the catchment area who are foreign-born, Rudan has received very few patients with a migrant background in recent years.

The three-component intervention

For an overview of the three-component intervention and the effect and process evaluation, see Figure 1.

For peer review only

SAM-TINA has three components:

Component 1: A health communication intervention targeting parents of children/youth with a migrant background.

Component 1 in detail:

The health communication intervention will be delivered in one session by health communicators, either at a venue (see 'Planning and preparations for Component 1') or digitally (in line with restrictions due to the Covid-19 pandemic). The health communication sessions are offered to parents of children and youth with a migrant background and are delivered over a period of 6-12 months. Each session is approximately 2.5 hours long, with a minimum and maximum of 2-3 and 8 participants, respectively. Sessions are delivered in participants' mother tongue (Arabic, Somali, Tigrinya, Amarinya, or Farsi), in simple Swedish, or with an interpreter. Sessions are offered primarily in languages deemed most common in the community by staff members at venues hosting the sessions. Sessions in other languages are offered upon active interest from participants.

Planning and preparing for Component 1:

In 2019-2020, the research group and Transkulturellt Centrum worked to create partnerships with units of the municipality and non-profit organisations in Haninge. Suitable hosts and venues for were identified with the help of the municipality's coordinators for refugee integration and parental support, "Swedish for Immigrants" classes in Haninge, parental support groups run by the local municipality targeting newly-arrived parents, toddler-parent groups, and the Swedish Church. Recruitment, group scheduling, and later Covid-19 adjustments were planned in joint meetings between these stakeholders, Transkulturellt Centrum, and SAM-TINA researchers. Additional venues may be added during the study.

Workshops with health communicators at Transkulturellt Centrum aimed to increase their knowledge of child mental health and the organisation of mental health services. These workshops were led by an experienced child psychologist and child psychiatrist. In addition, a specific tool kit was developed, in Swedish, consisting of PowerPoint slides with discussion points and case discussions, covering six chapters:

I. Normal development compared with signs of mental distress amongst children and adolescents
II. Parenting skills
III. Potential barriers to care from a migrant perspective
IV. Available mental health services and care levels
V. Services at Rudan and the role of a psychologist
VI. How to contact Rudan

The content of this toolkit was informed by the expertise and experience of professionals who have contact with our target population, including health communicators themselves. Health communicators can then adapt the toolkit to the needs of each specific group (in terms of language, for example).

Evaluation measures of Component 1

- Follow up questionnaires will be distributed before, directly after, and three months after the intervention by post, e-mail, or by hand to parents who have signed up for the sessions and consented to be contacted. In the absence of a validated instrument, this questionnaire was created for SAM-TINA. All questionnaires have been translated to the language the session is delivered in.
- A questionnaire for patients visiting Rudan for the first time will be given out to all new visitors, by email or by hand. The questionnaire explores why visitors chose to seek help at

Rudan and how they accessed the service. For patients younger than 14 years old, a caregiver, if present, will be asked to fill in the questionnaire.
- Analysis of register data from multiple registers, including the Psychiatry Sweden database, comparing our target population with non-migrant children (https://ki.se/en/gph/epidemiology-of-psychiatric-conditions-substance-use-and-social-environment-epicss) as done in [5-7], see 'The effect evaluation of the three-component intervention'.
- Focus group interviews, see 'The process evaluation of the three-component intervention'.

Component 2: Training for professionals and volunteers (herein referred to as TINA-facilitators) who potentially have contact with parents and children with a migrant background, in order to increase referrals to Rudan.

Component 2 in detail:

Training of TINA-facilitators is implemented and moderated by Transkulturellt Centrum's staff (except for the volunteer training, which is organised by the research group). The course length and format varies, depending on participating organisation's ability to allow staff to attend. All training is delivered digitally to minimise the spread of Covid-19. Most are one-day sessions but can be two half-day, or three short, sessions. Online training for pre-school teachers, with pre-recorded material, is accessible for 4-6 weeks.

Planning and preparing for Component 2:

The research group mapped the demographics of Haninge municipality (see 'Site'), including mapping the number of children with a migrant background by residential area, in order to identify priority neighbourhoods for the intervention. Four neighbourhoods were identified. In 2019-2020, the research group and Transkulturellt Centrum worked to recruit healthcare, municipality, and community organisations in Haninge. We primarily invited units serving children in the four areas to participate in the training intervention. To tailor the content, format, and duration of the training to the diverse needs of the participating units, nine group interviews were conducted. Based on the results of these interviews, and Transkulturellt Centrum´s previous experience with providing staff training, seven separate training programs were designed. All are a minimum of four hours with a common core of lectures (see below), based upon the interviews with professionals on their training needs and the migrant experience:

I. How to identify mental distress among children and adolescents
II. Potential mental health consequences of migration
III. Barriers to care for migrants in Sweden and how this can differ between groups
IV. Mental health services at Rudan

Moreover, lectures were added to cover specific needs of the individual units, such as how to differentiate learning disabilities from post-traumatic stress for the school healthcare staff. After the training, all participants will be offered information, digitally or on paper, about Rudan (in Polish, Russian, Arabic, Turkish, Spanish, Somali, Tigrinya, English, French, Persian/Dari, and Swedish) to distribute to eligible families.

Evaluations measures of Component 2

- Questionnaires, including short-term effect and process outcomes, will be sent by email to TINA facilitators who signed up for the training and have consented to be contacted before, directly after, and three months after the training. These questionnaires are a combination of a modified version of a questionnaire previously used in the region for similar purposes [13], and items on readiness for change from the implementation outcome measure (IPM) [14].

For peer review only

They also include questions from a previously used survey [15] to measure learning and reactions to evaluate the training experience.

- A similar, shorter version of the Component 2 questionnaire has been developed for the volunteer training.
- The questionnaire to first-time patients at Rudan, see 'Evaluations measures of Component 1'.
- Analysis of register data, see 'Evaluations measures of Component 1'.
- Semi-structured interviews, see 'The process evaluation of the three-component intervention'.

Component 3: Increasing access to care at Rudan through various strategies to lowering barriers to seeking care.

Planning and preparing for Component 3:

Interviews with directors and staff at primary care-based mental health services for children were conducted to explore barriers to care and support given to migrant parents (published in Swedish) [16]. The research group then worked with Rudan's manager and the head of the primary healthcare centre to anchor the project, and a project coordinator was appointed at Rudan. Through discussions with the project coordinator, staff at the primary health centre, Rudan's psychologists, and Transkulturellt Centrum's health communicators, the research group suggested the following interventions to lower barriers to care for migrant families:

- Written information (leaflets, posters, and business cards) about Rudan available in the primary healthcare centre waiting area and on its website (https://www.aleris.se/har-finns-vi/stockholm/aleris-rudans-vardcentral/psykosocial-mottagning-barn-och-ungdom/) in Polish, Russian, Arabic, Turkish, Spanish, Somali, Tigrinya, English, French, Persian/Dari, and Swedish.
- A direct telephone number for appointment booking, without a phone menu, available from March 2021. The phoneline is manned from 14:00-15:00 during the clinic's regular opening hours. Messages left outside of these hours will be listened to once a day and individuals contacted.
- Appointment booking drop-in service at Rudan's reception, available from March 2021 between 14:00-15:00 during regular opening hours.
- A tablet available in Rudan's reception, with translation provided by the app Care-to-Translate (https://caretotranslate.com/sv/), for appointment booking from April 2021.
- A leaflet sent out to all new patients at Rudan, in the child's or parent/caregiver's preferred language, together with appointment details and containing information on what to expect from their first visit. Available from June 2021.

Evaluations measures of Component 3

- Questionnaire to first-time patients at Rudan, see 'Evaluations measures of Component 1'.
- Analysis of register data, see 'Evaluations measures of Component 1'.
- A daily log, kept by the project coordinator, to document any implementation issues.
- Interviews with Rudan's staff to explore challenges in the implementation process and to understand barriers and facilitators for implementation.

<u>Time frame:</u>

The three components are implemented in parallel in order to create a synergistic effect:

Component 1: Trainings are organised over a period of 5.5 months (start date: Nov 11th 2020, planned completion: May 30th 2021).

For peer review only

Component 2: Health communication sessions will be offered over a 6–12-month period (planned start in Nov 2020 was delayed until Feb 2021).

Component 3: Interventions will be implemented over a 12-month period from March 2021.

Recruitment and eligibility

Component 1: Health communication interventions targeting parents of children/youth with a migrant background.

Recruitment of participants

Recruitment of parents is currently ongoing. Information in multiple languages is being disseminated to parents by health communicators and hosts at the venues (see 'The three-component intervention'). In addition, short video clips in Arabic, Tigrinya, Somali, Farsi, and easy Swedish have been developed for the project and will be disseminated online. They can be viewed at the project homepage (https://ki.se/gph/informationstraffar-for-foraldrar-i-haninge).

Eligibility

All parents to minors are eligible to take part, independent of country of birth, length of stay in Sweden, or language. However, a minimum of 2-3 participants are required for a session, which could lead to exclusion of parents with a less commonly spoken preferred language. All participants are welcome in the sessions, independent of literacy level, but illiterate participants are excluded from the follow-up evaluations due to the nature of the questionnaire.

Component 2: Training of TINA-facilitators who potentially have contact with parents and children with a migrant background, to increase the number of referrals to Rudan.

Recruitment of participants

Recruitment for the TINA facilitator training for professionals was achieved mainly with the help of key staff members, unit directors, and posts on the municipality's intranet. Some of the training programs were advertised in Transkulturellt Centrum's overall training program which reaches healthcare staff within the Stockholm Region. The recruitment of participants for the TINA -facilitator training for volunteers engaged in civil society with Haninge is still ongoing, assisted by key stakeholders and contact with community organisations.

Eligibility

Individuals who are expected to have contact with children, adolescents, and families with a migrant background are eligible to participate. This includes staff from the primary healthcare centres, paediatric clinics, adolescent health and addiction centres, social services, pre-schools, schools, and non-profit organisations (such as sports teams). Units who serve the populations in the four prioritised neighbourhoods (see 'Site') were prioritised for recruitment.

Component 3: Increasing access to care at Rudan through various strategies to lowering barriers to seeking care at this service.

Recruitment of participants and eligibility

All children under 18 years of age who have themselves, or both of their parents, been in Sweden for less than ten years and who have visited the primary healthcare centre are eligible to participate.

The effect evaluation of the three-component intervention

An effect evaluation aims to establish the causal association between an intervention in the target population and its intended outcomes.

For peer review only

Primary aim

The primary aim of the effect evaluation is to assess if the three components together increase the number of children and youth with a migrant background seeking care at Rudan for mild to moderate mental health problems for the first time.

Research questions

1. Will the observed rate of children seeking care at Rudan for the first time exceed the expected during the 12 months following the start of the intervention (exposed period), based on the rate of access in the 24 months preceding the intervention (unexposed period), and is there a difference between children and youth with and without a migrant background?
2. Will there be a change in the demographic composition of children seeking care at Rudan following start of the intervention (e.g. own or parents´ country/region of birth, time in Sweden, migrant status, and age)?
3. Will the observed rate and demographic composition of children seeking care at Rudan for the first time during the 12 months following the start of the intervention, based on the rate in the 24 months preceding the intervention, differ as compared to other primary care-based mental health services in the Stockholm Region, and is there a difference between children and youth with and without a migrant background?

Outcome measure

Number of first visit consultations at Rudan (onsite and digital) before and 3-, 6-, and 12-months following the start of interventions.

Secondary aim

The secondary aim of the effect evaluation is to investigate if the three individual components work as they were intended to.

Research questions

a. Will there be a change in the self-perceived knowledge and attitudes among staff and/or volunteers exposed to the training component? (e.g. increase in knowledge of signs of mental distress, change in attitudes towards working with families with a migrant background)?
b. Will the observed rate of referrals to Rudan from units exposed to the training exceed the expected during the 12 months following the start of the training component, based on the rate of access in the 24 months preceding the intervention), and differ compared to unexposed units?
c. Will there be a change in the self-perceived knowledge and attitudes among parents exposed to the health communication component (e.g. increased knowledge of mental health)?
d. What are the self-reported pathways to seek the service among first-time patients at Rudan following the start of the intervention?
e. Will there be a change in perceived barriers to connect with the service, among first-time patients at Rudan following start of the Component 3?

Outcome measures

- Staff and volunteer self-reported knowledge and attitudes at baseline, directly after, and 3 months following the training component (measured using the questionnaires for staff and for volunteers).

For peer review only

- Parents self-reported knowledge and attitudes at baseline, directly after, and 3 months following the health communication component (measured using the health communication questionnaire).
- First-time patients self-reported pathways to Rudan and self-perceived barriers to seek care at the service (using the first visit questionnaire).
- Number of referrals to Rudan from units exposed to the training component at baseline and 3-, 6-, and 12-months following start of the training (measured using data extracted from registers, see 'Primary aim: outcome measures' and from the first-visit questionnaire).

Methods and statistical analyses

For the primary aim we will analyse changes over time in children seeking care at Rudan and other primary care-based services in Stockholm, before and after the start of the intervention using the linked register data (as done in [5-7]) in a time-series design, where the outcome will be explored in one-month units, using Poisson regressions. Data from children from a non-migrant background will be used as a control. This method will also be used to analyse one of the secondary aims: referrals to Rudan. For the other secondary aims we will analyse the questionnaires using multiple logistic regressions models in both cross sectional and longitudinal designs.

<u>The process evaluation of the three-component intervention</u>

The process evaluation aims to facilitate an understanding of the effects of the intervention and provide information about under which circumstances a complex public health intervention to increase help-seeking among migrants can be implemented. The process evaluation will focus on assessing implementation fidelity (i.e. adherence to the intervention, and moderating factors that may influence the degree of fidelity) using a modified version of the Conceptual Framework for Implementation Fidelity (CFIF)[17].

Data collection and participants

A mixed methods approach will be applied, including questionnaires, semi-structured interviews, focus group discussions, and diaries. Interview guides will be developed based on the guiding framework, CFIF [17]. The interviews and focus group discussions will be conducted and reported following the COREQ checklist [18]. The data collection for each intervention component is outlined below and in Table 1.

Component 1: A health communication intervention targeting parents of children/youth with a migrant background.

Questionnaires at baseline, directly after, and three months after the health communication session will be used to assess satisfaction, usefulness, and benefits of the sessions, as well as to understand the demographics of parents who were recruited and attended the sessions.

We will perform focus group discussions with health communication participants to obtain a deeper understanding of their perceptions of these sessions. These groups will be conducted participants' preferred language (Arabic, Dari, Somali, or Tigrinya), and the interview guide will be translated from Swedish to this language before back-translation to Swedish to check for accuracy. Recruitment of participants for the focus group discussions will be conducted by the key actors at the venues recruiting participants to the health communication. A purposeful sampling approach will be used to ensure variation in the sample (including participants from different countries). The focus group discussions will be conducted in parallel to the health communication sessions and will be ongoing throughout this component of the intervention.

Component 2: Training of TINA-facilitators who potentially have contact with parents and children with a migrant background, to increase the number of referrals to Rudan.

For peer review only

The training of professionals will be evaluated by combining data from distributed questionnaires at baseline, directly after, and three months after the training, as well as interviews. Attendance lists will be used to collect information about the number of recruited participants and what proportion of target group participated in this component (coverage).

Semi-structured interviews will be conducted to assess participants' satisfaction with the training, whether the training led to behaviour changes, and potential moderating factors to implementation of what was learned from the training at the workplace. To understand contextual and organisational differences, a purposive sampling approach will be applied to recruit informants from different work units and professional groups. During the training, participants are asked to reflect on how to transfer knowledge gained during the training to their practice, and to concretise this into a plan for how to identify and refer children with mental health problems to the correct care level. The evaluation team will follow up on whether this plan has been created and implemented by contacting an assigned "contact person" at the participating units. Contact persons will be interviewed and, if necessary, more participants will be invited for interviews to assess the aforementioned implementation outcomes.

Component 3: Increasing access to care at Rudan by various strategies to lower barriers to seeking care.

A notebook will be used by staff at Rudan to document foreseen and unforeseen events related to the changes put in place related to the third intervention component. The notebook is a register of events and processes that take place over time. It will also be used for documenting problems that arise, and to note impressions and experiences of this process.

Data Analysis

The use of mixed methods will allow quantitative and qualitative combination of data collection sources, which can validate the findings and give a deeper understanding of the implementation process.

- Qualitative analysis

All interviews and focus group discussions will be audio recorded and transcribed verbatim. The transcripts will be analysed using deductive content analysis [19] based on the guiding framework CFIF [17]. Information documented in the diary is an additional qualitative data source. Reported expected and unintended events during the period of implementation will be compiled and added to the analysis.

- Statistical analysis

For survey data, statistical analyses will be used to measure changes over time within the groups of training participants and health communication participants. Data on moderating factors and fidelity will be used to predict intervention outcomes (for example, increased knowledge as assessed in the effect evaluation). Survey answers will be dichotomised (yes/no) and logistic regression or mixed logistic regression models will be used to explore possible variation over time. As sensitivity analyses, ordinal logistic regression will be performed to test the association for ordinal outcomes as a 5-score scale. Possible heterogeneity among health centres will be taken into account using multilevel models.

<u>Methodological limitations</u>

SAM-TINA is resource-intensive, so a cost-benefit analysis would have been beneficial but was not feasible. Furthermore, a lack of available instruments to evaluate complex interventions meant that the research group developed the survey tools themselves. It would have been beneficial to involve the target population in the design of the health communication intervention, but the content was

For peer review only

instead informed by interviews with professionals who have contact with this group. Finally, the fact that the intervention included just one municipality may limit the generalisability of findings to other contexts.

Patient and Public Involvement

Haninge municipality and non-profit organisations within Haninge were involved in the research from the planning stage (see 'Planning and preparing for Component 1-3'). Their involvement helped us to plan and design the components but not in their evaluation; this was carried out solely by the research group (with the exception of testing the pilot versions of the questionnaires). The findings will be disseminated to relevant stakeholders within Haninge municipality and nationally.

Ethics and Dissemination

All planned studied are approved by the Swedish Ethical Review Authority dnr 2017-135-31/5, 2019-06275, 2020-03640, 2020-06341, 2020-03642 and 2020-04180.

Informed consent will be obtained from all participants. The results of the project will be published continually in peer-reviewed scientific journals (four articles planned) and presented at conferences over a minimum period of eight years. Beyond academic output, the findings of the SAM-TINA project will be disseminated to relevant stakeholders within Haninge municipality and nationally.

Author Contributions

CD and ACH conceived the project. VP, together with ACH, KA, and AH, planned the manuscript's content and structure. VP and EG wrote the Introduction, while ACH, KA, HA, and AH wrote the Methods and analysis. VP was responsible for compiling these sections into a complete manuscript with supervision from ACH, who critically revised the manuscript at all stages. All authors reviewed the study findings and read and approved the final version before submission.

Funding

This work was supported by Swedish research council (Vetenskapsrådet) grant number: 2018-05763.

ACH was funded by Forte grant number: 2016-00870.

Competing interests

None declared.

Figure Legends

Table 1. The general process evaluation plan including general process outcome questions, areas to measure, and data sources.

Figure 1. A schematic overview of the three component intervention and the effect and process evaluation.

References

1. Kouider EB, Lorenz AL, Dupont M, Petermann F. Anxiety disorders in migrant and nonmigrant children: Risk factors and treatment success. *Kindh Entwickl*. 2015;24:252-262
2. Barghadouch A, Kristiansen M, Jervelund SS, Hjern A, Montgomery E, Norredam M. Refugee children have fewer contacts to psychiatric healthcare services: An analysis of a subset of refugee children compared to danish-born peers. *Social Psychiatry and Psychiatric Epidemiology*. 2016;51:1125-1136

For peer review only

3. Lindstrom M, Moden B, Rosvall M. Country of birth, parental background and self-rated health among adolescents: A population-based study. *Scandinavian Journal of Public Health*. 2014;42:743-750
4. Bronstein I, Montgomery P, Dobrowolski S. Ptsd in asylum-seeking male adolescents from afghanistan. *J Trauma Stress*. 2012;25:551-557
5. Hollander AC, Mackay E, Sjoqvist H, Kirkbride JB, Baarnhielm S, Dalman C. Psychiatric care use among migrants to sweden compared with swedish-born residents: A longitudinal cohort study of 5 150 753 people. *BMJ Glob Health*. 2020;5
6. Sirén A, Wicks S, Lindberg L, Dalman C. Uppföljning av första linjen psykisk ohälsa barn och unga 2018
7. Axelsson L, Baarnhielm S, Dalman C, Hollander AC. Differences in psychiatric care utilisation among unaccompanied refugee minors, accompanied migrant minors, and swedish-born minors. *Soc Psychiatry Psychiatr Epidemiol*. 2020;55:1449-1456
8. Manhica H, Almquist Y, Rostila M, Hjern A. The use of psychiatric services by young adults who came to sweden as teenage refugees: A national cohort study. *Epidemiol Psych Sci*. 2017;26:526-534
9. Bjorkenstam E, Helgesson M, Norredam M, Sijbrandij M, de Montgomery CJ, Mittendorfer-Rutz E. Differences in psychiatric care utilization between refugees, non-refugee migrants and swedish-born youth. *Psychol Med*. 2020:1-11
10. Place V, Nabb B, Assel K, Åhlen J, Gubi E, Hagström A, et al. Perceived barriers to care for migrant children and young people with mental and neurodevelopmental health problems in high-income countries: A meta-ethnography. Submitted
11. Fazel M, Reed RV, Panter-Brick C, Stein A. Mental health of displaced and refugee children resettled in high-income countries: Risk and protective factors. *Lancet*. 2012;379:266-282
12. Place V, Nabb B, Viksten Assel K, Baarnhielm S, Dalman C, Hollander AC. Interventions to increase migrants' care-seeking behaviour for stigmatised conditions: A scoping review. *Soc Psychiatry Psychiatr Epidemiol*. 2021
13. Baarnhielm S, Edlund AS, Ioannou M, Dahlin M. Approaching the vulnerability of refugees: Evaluation of cross-cultural psychiatric training of staff in mental health care and refugee reception in sweden. *BMC Med Educ*. 2014;14:207
14. Randall R, Nielsen K, Tvedt SD. The development of five scales to measure employees' appraisals of organizational-level stress management interventions. *Work Stress*. 2009;23:1-23
15. Mosson R, Augustsson H, Back A, Ahstrom M, Schwarz UV, Richter A, et al. Building implementation capacity (bic): A longitudinal mixed methods evaluation of a team intervention. *Bmc Health Services Research*. 2019;19
16. Åhlén J, Dalman C. Barn och ungdomar med migrationsbakgrund i första linjen för psykisk ohälsa: Tillgänglighet till vård, tillgång till översatta arbetsinstrument, och verksamhetens struktur. En rapport inom TINA-projektet. 2020
17. Hasson H. Systematic evaluation of implementation fidelity of complex interventions in health and social care. *Implementation Science*. 2010;5
18. Tong A, Sainsbury P, Craig J. Consolidated criteria for reporting qualitative research (coreq): A 32-item checklist for interviews and focus groups. *Int J Qual Health C*. 2007;19:349-357
19. Hsieh HF, Shannon SE. Three approaches to qualitative content analysis. *Qualitative Health Research*. 2005;15:1277-1288

For peer review only

Table 1. The general process evaluation plan including general process outcome questions, areas to measure, and data sources.

Process evaluation objective	Overarching research questions	Data source and data collection
**Adherence**		
**Content**	Were the three intervention components implemented as planned?	Questionnaires, interviews, diary
**Coverage**	What proportion of the targeted groups participated in the intervention?	Attendance lists
**Moderating factors**		
**Recruitment**	What barriers for maintaining involvement of individuals/groups in the intervention was identified?<br>What recruitment procedures were used to engage professionals and parents in the intervention?	Meeting minutes, interviews, surveys
**Participant responsiveness**	Did participants find intervention content useful?<br>How satisfied were the participants with the intervention services?<br>How did the participants perceive the relevance of the intervention?	Questionnaires, interviews
**Strategies to facilitate implementation**	Which strategies were put in place by work units participating in the training of professionals to implement learnings from the training?	Interviews
**Context (inner and outer)**	What factors at policy, organisational and<br>work group levels affected the implementation?<br>Which barriers and facilitators to delivery as planned can be identified?	Interviews, Meeting minutes, Diary
**Quality of delivery**	How did professionals perceive the impact of the intervention e.g., which elements did they find to be most helpful and unhelpful in meeting their needs?	Interviews
**Intervention complexity**	How complex is this intervention?	A group of external researchers will evaluate the intervention complexity

Modified from Hasson H. Systematic evaluation of implementation fidelity of complex interventions in health and social care [17].

For peer review only

The Intervention

Component I: Health Communication

- Health communication to small groups of parents of children/youth with a migrant background
- Delivered digitally or on-site in various languages over a 6-month period

Component II: Training of TINA-facilitators

- Transkulturellt Centrum (TC) will provide training to professionals and volunteers who potentially have contact with families with a migrant background
- Delivered digitally over a 6-12 month period

Component III: Lowering barriers to care at Rudan

Interventions to lower barriers to care at a primary care-based mental health service (Rudan) over a 12-month period:

- Written information about Rudan, available in the waiting area and online, in various languages
- Direct telephone number for appointment booking
- Appointment booking drop-in service
- Appointment booking, with a tablet device and translation by the app Care-to-Translate, at Rudan
- Written information sent out with appointment details to new patients in their preferred language

Data Collection

Health Communication

Effect & Process

- Questionnaires to parents before (T0), directly after (T1), and three months after (T2) the health communication intervention

Effect

- Questionnaire to first-time patients at Rudan exploring facilitators and perceived barriers to care-seeking
- Linked health care register data

Process

- Focus group discussions with health communication participants

Training of TINA-facilitators

Effect & Process

- Questionnaires to staff and volunteers before (T0), directly after (T1), and three months after (T2) training

Effect

- Questionnaire to first-time patients at Rudan exploring facilitators and perceived barriers to care- seeking
- Data on referrals extracted from the Take Care medical records system
- Linked health care register data

Process

- Semi-structured interviews with TINA-facilitator training participants

Lowering barriers to care at Rudan

Effect

- Questionnaire to first-time patients at Rudan exploring facilitators and perceived barriers to care-seeking
- Linked health care register data

Process

- A daily log to document the implementation process, kept by Rudan staff
- Interviews with Rudan staff to explore barriers and facilitators for implementation

Planned Data Analyses

- Survey and register data will be analysed using multiple logistic regression models to analyse changes over time
- Interviews, focus group, and daily log data will be analysed using deductive content analysis
